# Bacterial Profile, Antimicrobial Susceptibility Pattern, and Associated Factors among Dental Caries-Suspected Patients Attending the Ayder Comprehensive Specialized Hospital and Private Dental Clinic in Mekelle, Northern Ethiopia

**DOI:** 10.1155/2022/3463472

**Published:** 2022-10-17

**Authors:** Abu Kiros, Muthupandian Saravanan, Selam Niguse, Dawit Gebregziabher, Getahun Kahsay, Ranjithkumar Dhandapani, Ragul Paramasivam, Tadele Araya, Tsehaye Asmelash

**Affiliations:** ^1^Department of Medical Microbiology and Immunology, Division of Biomedical Sciences, School of Medicine, College of Health Sciences, Mekelle, Ethiopia; ^2^AMR and Nanomedicine Laboratory, Department of Pharmacology, Saveetha Dental College, Saveetha Institute of Medical and Technical Sciences (SIMATS), Chennai 600 077, India; ^3^Research and Development Division, Chimertech Private Limited, Chennai, India

## Abstract

**Background:**

Dental caries is a major public oral infectious disease globally due to its high prevalence and significant social impact. Many studies have been conducted on dental caries in Ethiopia; however, they fail to convey the antimicrobial resistance in the oral environment.

**Objective:**

This study was conducted to determine the antimicrobial susceptibility patterns and biofilm formation in the bacteria isolated from dental caries and its associated factors of dental caries in THE Ayder Comprehensive Specialized Hospital and private dental clinics located at Mekelle, Ethiopia.

**Methods:**

A cross-sectional study was conducted from September 2019 to October 2020. Sociodemographic characteristic, behavioral, and clinical data were collected using structured questionnaires. A total of 422 dental caries-suspected patients were selected and coronal caries scraps were collected by the dentist aseptically; these samples were transported to a microbiological laboratory to identify the antibiotic sensitivity assay and biofilm formation by the isolated pathogens. The data was analyzed using SPSS version 22. The *P* value of ≤0.05 was considered statistically significant.

**Results:**

The overall prevalence of culture-positive samples was found to be 196 (46.4%). From the 196 culture-positive samples, 327 bacteria were isolated. Out of 327 bacterial isolates, 196 (46.4%) were identified as *Streptococcus mutans* and 69 (35.2%) were identified to be *Staphylococcus aureus.* From the isolated bacteria, 311 (95.1%) organisms were identified as positive for biofilm formation. From the AST assay, we have identified that penicillin has the highest resistance rate of 76.5%, followed by tetracycline at 64.8%. In contrast, the antibiotics such as cefoxitin and chloramphenicol have a sensitivity of 83.5% and 81.6% to all the bacterial isolates. The overall prevalence of multidrug resistance (MDR) in the isolates was found to be 40.4%. With respect to the associated risk factors, the white spot (AOR = 3.885, 95% CI 1.282-11.767, *P* = 0.016), gum bleeding (AOR = 2.820, 95% CI 1.006-7.907, *P* = 0.049), toothache (AOR = 2.27, 95% CI 0.58-0.885, *P* = 0.033), and chocolate consumption (AOR = 5.314, 95% CI 1.760-16.040, *P* = 0.003) were statically associated with dental caries bacterial infection.

**Conclusion:**

Based on our findings, we recommend the integration of routine culture and AST into clinical practice that might support the diagnosis and management of MDR in dental caries. The education on proper dietary habits might support the prevention and control of dental caries. It is important to provide health education on how to improve oral health in the study area. The education on proper dietary habits might support the prevention and control of dental caries. Further study is needed to find the other determinant factors of dental caries.

## 1. Introduction

Dental caries is the localized destruction of susceptible dental hard tissues by acidic by-products from bacterial fermentation of dietary carbohydrates [1, 2]. Dental caries is a major public oral infectious disease globally due to its high prevalence and significant social impact. Dental caries result from an ecological imbalance in the equilibrium between tooth minerals and oral biofilm [3, 4]. Dental caries is the biofilm-induced disease that can affect any age group and is highly related to and influenced by the patient's dietary habits, frequent sweet food intake. These factors, together with time, promote the microbial residence in the accumulated dental plaque to initiate dental caries infection [5]. Oral biofilm is a three-dimensional complex structure of different microorganisms inhabiting the oral cavity; if remained for a long time without treatment or interventions, biofilm can undergo maturation leading to dental caries development. It is believed that *Streptococcus mutans* plays an important role in forming multidimensional and complex structures on oral mucosa and tooth enamel [6]. Cells adhered to by biofilm are more resistant to conventional antibiotics compared to their planktonic ones. Bacterial extracellular polymeric substances (EPS) are strong barrier molecules that influence the rate of transport to the deep biofilm layer. In oral biofilm, bacteria are continuously challenged by changes in the environmental conditions. As a response to such challenges, *S. mutans* dominant in oral biofilms rely on the MDR transporter allowing withstanding toxic compounds produced by competing species or present in the plaque environment [7] [8]. According to the World Health Organization (WHO) reports, more than 90% of the population experiences caries, which is more prevalent in Asian and Latin American countries [9, 10]. In 2019, about 3.58 billion people have been assessed with dental caries infections [11]. Dental caries is the most chronic oral infectious disease in the world [12]. The policy of the WHO global oral health programme emphasizes that oral health is integral and essential for general health and the oral health is a determinant factor for the quality of life [13, 14].

The prevalence of dental caries was reported as 24.1% in Africa, 63.3% in Nigeria, 45.6% in China, and 65% in Kenya [15–19]. The main reason attributed for this high prevalence rate is because of a lack of knowledge, poor oral hygiene, and poor disease prevention methods [20, 21]. In the study conducted in Egypt, 74% prevalence of dental caries among children was identified [22]. In Ethiopia, 78.2% prevalence of dental caries was found in the patients attending the Debre Tabor General Hospital [23], 35.4% prevalence of dental caries was identified in the patients attending the Axum Primary School [24], and 68.7% prevalence of dental caries was found in the patients attending the Debre Berhan Referral Hospital [25]. Despite the fact that many studies have been conducted to study dental caries in Ethiopia, none assessed the bacterial profile of dental caries. Dental caries causes low to severe pain aggravated by chewing on any food substance, psychosocial disturbance, difficulty communicating due to missing or discolored teeth, and has the greatest impact on daily life activities [26]. However, if not treated early, it leads to severe intolerable pain, facial swellings, difficulty in chewing and swallowing, limited mouth opening, difficulty in breathing, and, in some cases, death [27]. Management strategies for tooth decay are adopted as surgical removal of the decayed tooth, and a more geometrically perfect cavity is created and filled with the most compatible and artificial materials. These materials might lead to secondary bacterial infection and bacteraemia [28, 29]. Nowadays, people spend huge amounts of money and time in treating dental caries. Therefore, it is essential to prevent and control dental caries [30].

The conventional methods to diagnose dental caries are based on the detection of visible color, texture change, tackling sensation using a dental explorer, and radiographs. However, radiographs are not useful for detecting early enamel caries. A microbiological approach of diagnosis would help in diagnosing early tooth decay. However, the implementation of this strategy is limited in resource-constrained settings. Therefore, generating evidence through research on the early screening for pathogenic bacteria and identifying their antimicrobial susceptibility test patterns are vital for infection prevention and implementers should consider the importance of this approach. In Ethiopia, there are few studies that show a higher prevalence of dental caries. However, the previous reports did not address the microbiological investigation and there is only one study done on the prevalence of *Streptococcus mutans* without assessing the AST assay to detect the AMR pathogens [25]. Moreover, there is no study on the bacterial profile in dental caries and the susceptibility pattern of the isolated bacteria [23–25]. Therefore, our study is aimed at determining the bacterial profile, antimicrobial susceptibility, biofilm formation in pathogens, and associated risk factors of dental caries in the study area. Our study will provide up-to-date information about the status of drug-resistant pathogens in the dental caries which could help the physicians select the best alternative drugs. Additionally, it could enroute the experts and policymakers to set the guidelines for treatments.

## 2. Materials and Methods

### 2.1. Study Area

The study was conducted at the dental clinic in ACSH and the union private dental clinics in Mekelle, northern Ethiopia. Mekelle town is the capital city of the Tigray regional state. Mekelle is located 787 km north of Addis Ababa, the capital city of Ethiopia, and covers an area of 109 square kilometers; its elevation is 2084 m above sea level. According to the 2007 census program, the total population of the town was reported to be about 258258. ACSH is the largest university hospital in the region and the second-largest hospital in Ethiopia. The ACSH dental clinic provides different services from dental care to different surgical procedures. According to the health management and information system of ACSH, an average of 5491 dental caries patients were treated in the year 2011. The inion special higher dental clinic, a pioneer of dentistry service in Tigray, was founded by Dr. Kidane Mamo Endale in 2002 at Kebelle along with 17 different branches. The clinic has a patient flow of about 1200/year.

### 2.2. Study Design: Inclusion and Exclusion Criteria

A cross-sectional study design was conducted from September 2019 to October 2020. All patients visiting the dental clinic of ACSH and the union private dental clinics were considered as a source of population. All patients suspected of dental caries who were attending a dental clinic in ACSH and the union private dental clinics were included in the study. All the age groups of people were included in this study. Patients who developed a complication and arrested dental caries were excluded from this study.

### 2.3. Sample Size Determination

The sample size was calcutated using single population proportion formula. The following parameters and assumptions were taken into consideration: *Z* = statistic for the level of confidence 95% (5%, *Zα*/2, 1.9 and 6) and the margin of error (*d*) = 5%. To our knowledge, there was no similar study previously done in Ethiopia; hence, we assume a 50% proportion (*P* = 0.5). Based on these assumptions, the actual sample size for the study is computed to achieve the following sample size.

(1) $$\begin{array}{c} n=\frac{{\left(Z\alpha /2\right)}^{2}P\;\left(1-P\right)}{d^{2}}, \end{array}$$

where *n* = sample size, *Zα*/2 = critical value = 1.96, *P* = proportion = 0.50, *d* = precision(marginal error) = 0.05.

The sample size was (1.96/.05)^2^ 0.5 (1 − 0.5) = 384. 10% contingency was 384 (10/100) = 38.4, 384 + 38.4, *n* = 422. Therefore, a total of *422* patients were included in the study.

### 2.4. Sampling Technique

**Figure 1:**
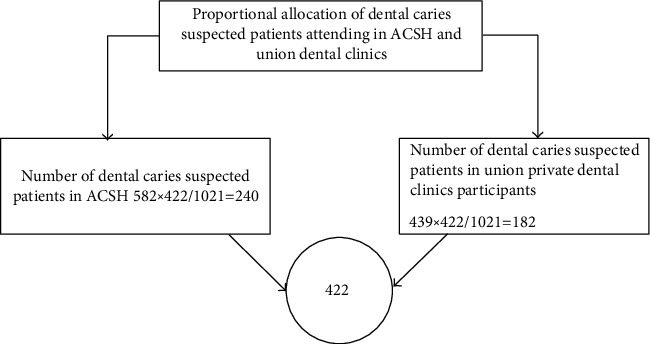
Schematic representation of the sampling procedure.

A consecutive convenience sampling technique was employed as shown in Figure 1 to include the study participants. The total patient flow within six months in ACSH was 582 and 439 were in the union private clinic.

### 2.5. Study Variables

We have considered the following dependent and independent variables: the bacterial profile, biofilm formation, and antibiotic susceptibility patterns were considered dependent variables. The sociodemographic variables such as age, sex, residence, occupation, and education; clinical factors such as poor oral hygiene, dental trauma, sensitivity to cold and hot, history of chronic disease, and history of dental procedure; behavioral factors such as oral hygiene practice, smoking cigarettes, sweet intake, tooth brushing, and chat chewing were considered independent variables.

### 2.6. Data Collection Procedures

Structured questionnaires were prepared to collect patient's sociodemographic (including age, sex, residence, occupation, and education), clinical (including poor oral hygiene, dental trauma, sensitivity to cold and hot, history of chronic disease, and history of dental procedure), and behavioral data (including oral hygiene practice, smoking cigarettes, sweet intake, tooth brushing, and chat chewing). Complete questionnaires could be found in the supplementary document (available here). The questionnaires were translated into the local language (Tigrigna) to generate quality and reliable data. The coronal caries scraps were collected using a sterile dental elevator and inserted into a sterile tube containing 2 mL normal saline. The tubes were labeled and transported to the medical microbiology laboratory. The samples are refrigerated at 2–8°C until further use.

### 2.7. Bacterial Isolation and Identification

The coronal caries scrap was vortexed for 30 seconds to become homogenized and then streaked on different agar plates such as Mitis Salivarius agar (HiMedia, India), blood agar, MacConkey agar, and Mannitol salt agar (Oxoid, Hampshire, UK) using a sterile cotton swab and incubated for 24 hours at 37°C. After incubation, colonies were observed for their morphology (opaque, spherical, and raised) and glistening bubbles on the surface due to excessive synthesis of glucan from sucrose on Mitis Salivarius agar media due to the growth of *S. mutans* [31][25, 32]. The isolated pure cultures were subcultured on a blood agar slant for further identification. Standard cultures including *E. faecalis* ATCC 29212, *E. coli* ATCC 25922, and *S. aureus* ATCC 25923 strains were obtained from the Tigray Health Research Institute.

### 2.8. Identifying Biofilm Forming Bacteria

The qualitative tube adherence method was used to detect the biofilm formation in the isolated bacteria [33]. Briefly, a loopful of the test organisms were inoculated in a test tube containing 10 mL of trypticase soy broth (TSB) medium (HiMedia, India) with 1 ml of 1% glucose and incubated for 24 hours at 37°C. After incubation, cultures were discarded and the tubes were left to dry. The dried tubes were stained with 0.1% crystal violet and subsequently washed with sterile deionized water to remove the excess stains. The positive biofilm-forming bacteria show the visible film on the side and bottom of the tube.

### 2.9. Antimicrobial Susceptibility Pattern of the Bacterial Isolates

Kirby-Bauer disk diffusion assay was employed to identify the AST. The turbidity of the isolate was set at 0.5 McFarland standards. The antibiotic disk such as tetracycline (TE) (30 *μ*g), doxycycline (DOX) (30 *μ*g), erythromycin (Ery) (15 *μ*g), clindamycin (CL) (10 *μ*g), penicillin (PE) (10 *μ*g), gentamicin (GE) (10 *μ*g), chloramphenicol (CAF) (30 *μ*g), ciprofloxacin (CIP) (5 *μ*g), cefoxitin (CEF) (30 *μ*g), trimethoprim-sulfamethoxazole (SXT) (1.25/23.75 *μ*g), and ceftriaxone (CEFT) (30 *μ*g) were used. Briefly, the Muller Hinton agar plates were swabbed with the test cultures and the antibiotic disk was placed on the plates and incubated at 37°C for 24 hours. After incubation, the zone of inhibition was measured and the results were recorded as sensitive (S), intermediate (I), or resistance (R) [34]. The observed results were interpreted according to the Clinical and Laboratory Standards Institute (CLSI) guidelines. Multidrug resistance (MDR) was defined as acquired nonsusceptibility to at least one agent in three or more antimicrobial categories [35].

### 2.10. Data Analysis Procedures

The collected data were entered, cleaned, and analyzed using Statistical Package for Social Sciences (SPSS) software version 22.0 (IBM, USA). Descriptive statistical analyses were computed using frequency, percentage, crosstabs, and mean and standard deviation. Binary logistic regression analysis was conducted to assess the presence and degree of association between dental caries and independent variables. The strength of association was presented by odds ratio, 95% confidence interval, and a *P* value of less than or equal to 0.05 on binary logistic regression; binary multiple logistic regression analyses were computed. The *P* value of less than or equal to 0.05 was considered a statistically significant association between risk factors and dental caries bacterial infection.

### 2.11. Ethical Clearance

The study was reviewed and approved by the Institutional Review Board (IRB) of CHS, MU. Support letters were obtained from respective concerned bodies. Informed consent or ascents were obtained after explaining the objective of the study to participants or guardians/parents, respectively. All the information collected through the study was kept confidential. The laboratory test results were communicated to the concerned clinicians.

## 3. Results

### 3.1. Characteristics of Study Participants

A total of 422 patients with dental caries were enrolled in the study, of which 227 (53.8%) patients were male. The majority 289 (68.5%) of the study participants were within the age group of 19–64 years while the lowest number 33 (7.8%) belonged to ≥65 years. Resident wise, 304 (72%) of the study participants were from urban areas and 118 (28%) of the study participants were from rural areas. Regarding the occupations, 186 (44.1%) of them were students and 65(15.4%) study participants were employed. From our study, majority of the study participants, 146 (34.6%), completed their primary education level and about 42 (9.9%) study participants have not taken formal education (Table 1).

### 3.2. Clinical Characteristics of Study Participants

**Figure 2:**
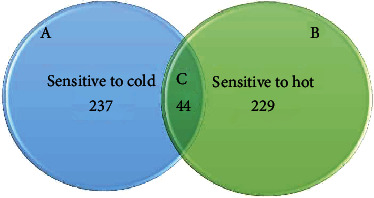
Venn diagram shows the study participant correlation between the (a) participants sensitive to cold, (b) participants sensitive to heat, and (c) participants sensitive to both hot and cold.

From the 422 study participants, 224 (53.1%) had a toothache, 207 (49.1%) had a white spot, 177 (41.9%) had gum bleeding, 237 (56.2%) were sensitive to cold, and 229 (54.3%) were sensitive to hot (Figure 2). About 145 (34.4%) participants previously visited dental clinics for dental procedures. Study participants who had a history of dental trauma were 95 (22.5%), and those who had a history of chronic disease were 57 (13.5%) (Table 2).

### 3.3. Behavioral Characteristics of Study Participants

In Table 3, we conclude that the majority of the study participants 351 (83.2%) brushed their teeth as a daily habit, and from those, 114 (27%) used toothpaste and 42 (10%) used charcoal, respectively. Among the participants, 95 (22.5%) were brushing their teeth in a circular motion and 84 (19.9%) were top to bottom motion, respectively. 100 (23.7%) participants cleaned their teeth before and after a meal and 74 (17.5%) participants cleaned their teeth once a week. 53 (12.6%) study participants were having the habit of chewing chat, and 78 (18.5%) participants were having the habit of smoking cigarettes; 347 (82.2%) of the participants have the habit of taking soft drinks. 351 (83.2%) of the study participants have the habit of sweet food consumption, and out of 351 participants, the majority of 105 (24.9%) participants consumed chocolate.

### 3.4. Prevalence of Bacterial Isolates among Dental Caries-Suspected Patients

*Streptococcus mutans* are Gram-positive cocci, a nonmotile facultative anaerobic microorganism that can metabolize carbohydrates and is considered the principal etiological agent of dental caries. Other pathogens were not considered members of the usual oral microbiota, including *Staphylococcus spp*., coagulase negative Staphylococci, and *Acinetobacter*, where these pathogens were detected in high-frequency dental caries [36]. We have isolated about 327 pure bacterial isolates from the collected samples, the predominant isolates were identified to be *Streptococcus mutans* 196 (46.4%) followed by *Staphylococcus aureus* which was found to be 69 (35.2%), and coagulase-negative *Staphylococci* (CoNS) was found to be 62 (31.6%). Out of 196 *Streptococcus mutans* cultures, 160 (81.6%) cultures were found to be a mixed bacterial culture, whereas 36 (18.4%) cultures were found to be pure conies (Table 4). The high prevalence of *Streptococcus mutans* in the urban participants were found to be 46.4% (141) and in the student participants of about 49.5% (92). This strain was mostly found in the age group below 18 years with 53% (53), and the participants who does not have any formal education was identified to be 72.7% (27). The prevalence of mixed isolates such as *Staphylococcus aureus* and *Streptococcus mutans* in urban participants were found to be 41.7% (62), this strain was mostly found in the age group between 19 and 64 with the 57.3% (55), the high prevalence of CoNS and *Streptococcus mutans* was found in the below 18 age category with 66% (35), these strains were mostly found in the female with 45.3% (29), the high prevalence of CoNS, *S. aureus*, and *S. mutans* was found in the rural population with 53.3% (24), and this strain is mostly found in the participants of the above 65 age group with 90.9% (10).

### 3.5. Identifying Biofilm-Forming Bacteria

**Figure 3:**
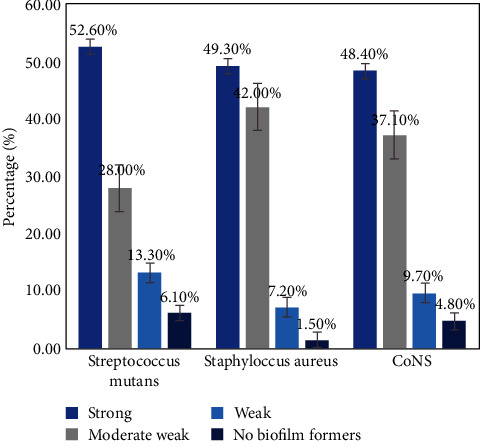
Biofilm detection by the collected bacterial isolates using the tube adhesion method.

The result of the qualitative tube adhesion method revealed that out of a total of 327 pure bacteria isolates, 311 (95.1%) isolates were recognized as biofilm producers, while the rest of the 16 (4.9%) isolates exhibited no biofilm formation capabilities. The biofilm producers with strong tube adherence were found to be 53.7% (167) bacteria, moderate biofilm formers were found to be 34.4% (107) bacteria, and weak biofilm producers were found to be 11.9% (37) bacteria. Among the isolates, 52.6% (103) were found to be *S. mutans*-strong biofilm producers than *S. aureus* which accounts for 49.3% (34) and CoNS which accounts for 48.4% (30) (Figure 3).

### 3.6. Association of Risk Factors among Dental Caries Patients

Binary logistic regression analysis was performed to assess the degree of association between independent and dependent variables. Multivariate logistic regressions were also performed for variables that showed a significant association with the dependent variables in the binary logistic regression analysis. Of 207 study participants, participants with white spots were found to be about 116 (56.0%) and these participants were more likely to have dental caries (AOR = 3.885, 95% CI, 1.282-11.767, *P* = 0.016) than those who did not have a white spot. Similarly, the participants who had gum bleeding were found to be 99 (55.9%) and these participants were susceptible to two times at risk for tooth decay (AOR = 2.820, 95% CI, 1.006-7.907, *P* = 0.049) than those without gum bleeding. Participants who had toothache were found to be 117 (52.2%), and these participants were three times at risk for developing dental caries (AOR = 2.27, 95% CI, 0.58-0.885, *P* = 0.033) than those who did not have a toothache. From our interpretation, 351 study participants consumed sweet foods; from the types of sweet foods, the participants consuming chocolates were identified as 65 (61.9%) and they are five times at risk for dental caries infection (AOR = 5.314, 95% CI, 1.760-16.040, *P* = 0.003) than those who did not consume chocolate (Table 5).

### 3.7. Antibiotic Susceptibility Patterns of the Bacterial Isolates

Table 6 summarizes the overall AMR profile of the Gram-positive bacteria. In this study, the sensitivity rates of the bacterial isolates ranged from 13.5% to 83.5% and the resistance rate of Gram-positive bacteria for antibiotics was between 3.4% and 76.5%. The highest resistance rate of 88.4% was observed in *S. aureus* against penicillin and 71% against ciprofloxacin. On the other hand, cefoxitin shows 81.9% sensitivity and chloramphenicol shows 73.9% sensitivity toward *S. aureus*. The isolated *S. mutans* (70.4% and 63.7.7%) show 70.4% resistance to penicillin and 63.7% resistance to tetracycline. On the other hand, 83.6% and 82.2% of *S. mutans* were 83.6% sensitive to cefoxitin and 82.2% sensitive to chloramphenicol, respectively. The isolated CoNS were 82.2% resistant to penicillin and 62.9% resistant to ciprofloxacin. However, they are 85.5% susceptible to cefoxitin and 87.1% sensitive to chloramphenicol (Table 6).

Multidrug resistance (MDR) was defined as acquired resistance to at least one agent in three or more antimicrobial categories according to the European CDC, 2015 [35]. All the bacterial species isolated from dental caries were resistant to one or more antimicrobial agents, and the overall MDR was found to be 40.4%. The multidrug resistance rate was high in *S. mutans* with 41.3% followed by *S. aureus* with 39.1% and CoNS with 38.7% (Table 7).

## 4. Discussion

There is limited data on dental caries, especially in the northern parts of Ethiopia. We attempted to assess the prevalence and associated factors of dental caries among patients attending the ACSH and union private dental clinics. This study revealed the overall prevalence of bacteria in dental caries as 196 (46.4%). The findings of our study were higher than those of the studies done in Nigeria (37.1%), Iraq, (36.2%), and Nepal, (40.3%) [15, 32, 37]. The difference might be due to increasing patterns of caries patients because dental caries is a chronic disease that progresses slowly in most people and every individual in the world is susceptible to dental caries once in their lifetime. However, this study was lower than studies conducted in Nepal by Yadav and Prakash which shows about 62.5% prevalence [38]. These differences might be due to different factors such as the study setting, sample size, sociocultural differences, and attitude. Supragingival plaque is dominated by Gram-positive bacteria including *Streptococcus mutans*, *Streptococcus sanguinis*, *Streptococcus mitis*, and *Streptococcus salivarius* [39–41]. *Streptococcus mutans* are the principal agent of enamel caries [38]. *Streptococcus mutans* are highly cariogenic, producing short-chain acids which soften hard tissues of the teeth and the ability to survive at low pH [42] and have three isozymes of glucosyltransferases catalyze and metabolize sucrose to synthesize insoluble extracellular polysaccharides, which increase their adherence to the tooth surface and persuade biofilm formation [43]. In the current study, the predominant isolate bacteria were *Streptococcus mutans* with 46.4%. The findings of this study were consistent with other studies conducted in Nigeria (45.6%) [16] and Nepal (43.7%) [38]. And the current study shows more Streptococcus mutant isolate than the previous studies conducted in India (22.8%), Nepal (40%) [30, 37], and Nigeria (18.7%) [15]. In other contrast, this finding was lower than the study done in Debre Birhan (68.7%) [25]. Other than *S. mutans* isolate, our study shows 35.2% prevalence of *Staphylococcus aureus* which is higher than other studies conducted in Nepal (31.6%) and Nigeria (28.9%) [15, 16, 38]. The difference might be due to the methodology, socioeconomic backgrounds, dietary behaviors, and differences in knowledge and practice regarding the tooth brushing habit.

In our study, the prevalence of mixed bacterial growth was 81.6%, which was comparable with the previous study done in Nigeria (86%) [15]. In contrast to another study, this finding was lower than the study done in Nepal (90%) [37]. Biofilm formations are one of the major virulence factors and facilitate its adherence and colonization. The diversity of the oral microbiota and the ability of oral microorganisms to form dental biofilm on the teeth, implants, and oral mucosal surfaces in a sophisticated manner have been characterized [44]. In this study, the predominant strong biofilm producer bacteria were *S*. *mutans* (52.6%), followed by *S. aureus* (49.3%), and CoNS (48.4%) which is comparable with a study done in Egypt with *S*. *mutans* (48.5%), *S. aureus* (53.2%), and CoNS (46.8%) [45]. However, this study was lower than the study done in Kenya where *S. aureus* has 59.8% prevalence [46]. The difference might be due to the difference in detecting the biofilm detection methods. Patients developing white spots were significantly associated with the risk of acquiring dental caries infection than those who did not have white spots. Poor dental hygiene results due to irregular tooth brushing habits which result in the formation of plaque as a consequence caused tooth decay. The finding of this study was in line with studies done in Debre Birhan, Debre Tabor, Aksum, Ethiopia, and Egypt [22–25, 47]. This indicates underutilization of dental health facilities; unhealthy dietary habits and gaps in the knowledge, attitude, and practice of dental hygiene were the major causes of poor oral hygiene to develop dental caries infection [48]. Gum bleeding was significantly associated with dental caries. Patients with gum bleeding were more likely to have dental caries than others who did not have gum bleeding. The gum bleeding increased the colonization of bacteria, and in severe cases, it causes the loss of a tooth. This might be an indicator of poor oral hygiene. These findings correlate with the previous studies [23–25].

The higher prevalence of dental caries in those who did not attend formal education than their counterparts could possibly be due to the indirect effect of education on dental caries. Those who attended formal education might have awareness of dental caries and take regular appropriate measures to prevent dental caries. An educated person can read and obtain information about oral health while those who are not educated may not know the cause of dental caries and the measures to be taken for its prevention. Moreover, information related to oral health might be given during formal education. Toothache was one of the major indicators of dental caries infection. The experience of tooth pain; the problem with eating, smiling, and communication due to missing; and discoloration have a foremost impact on people's everyday life [38]. In this study, patients who have toothache were 2.27 times more likely to have dental caries bacterial infection. This finding was supported by other studies done in Bahir Dar, Ethiopia, and Kenya [47, 49]. Consumption of sweet food like chocolate has been significantly associated with dental caries. The finding was in agreement with studies done in Bahir Dar, Finote Selam, Ethiopia, Egypt, and Zimbabwe [21, 22, 47, 50]. This might be associated with plentiful acid production by cariogenic bacteria that are adherent to the tooth as a consequence of the fermentation of sweet foods. Later on, the enamel of the hard teeth went into tooth decay [1, 2]. Treatment of dental infections depends on whether it is a low-level local infection or a severe infection of the fascial spaces. If possible, removal of the source of infection is the most important step in treating dental infection [51, 52]. Furthermore, it depends on the extent of dental caries and can range from the insertion of restorative material (filling) to tooth extraction. Irreversible pulpitis treatment includes root canal and extraction, and there is insufficient evidence to recommend antibiotics. A periapical abscess can complicate pulpitis. An uncomplicated periapical abscess is treatable with incision and drainage only. Periapical abscess complicated by systemic symptoms, cellulitis, or immunocompromised patients should receive antibiotics in addition to drainage [53]. Antibiotic therapy for dental infections is necessary for systemic symptoms, fascial space infections, and infections that spread to the bony cortex and surrounding soft tissue. Antibiotics such as amoxicillin, clindamycin, tetracycline, and erythromycin are the most common medication prescribed for dental infections [54–56].

In our study, we used the antibiotics such as tetracycline, erythromycin, and penicillin antibiotics to detect the antibiotic susceptibility test and found that the isolated bacteria were resistant to most of the used antibiotics. The overall prevalence of multidrug-resistant pathogens was identified to be 40.4%. This study's finding was comparable with other studies [37, 54]. The main reasons for multidrug resistance were because of the misuse of drugs. Caries can be prevented through perfect oral hygiene [45]. Many mechanical methods can be employed for this purpose. Among those, tooth brushing is the most frequently advocated and most widely exercised. The horizontal vibrating method also known as the bass method stresses the removal of plaque in the gingival sulcus and the interproximal spaces [34]. Minimizing the frequent consumption of more carbohydrates during night time is greatly helpful to prevent the formation of dental caries [57]. The burden of dental caries can be reduced by providing proper health education on how to keep oral hygiene and regular visit to a dental clinic.

## 5. Limitation

The major limitation of the present study is that the strictly anaerobic bacteria from the collected coronal caries scraping has not been isolated. Sweet food items and drinks were assessed by the usual patterns of intake, but the amount and the duration of intake were not assessed. The difficulty of radiological examination due to the lack of instruments and laboratory setup might reduce the actual magnitude of the problem. The monthly income of participants was not assessed. The determinant factors were not exhaustive. There could be other determinant factors of dental caries that our study did not address.

## 6. Conclusion

The overall prevalence of dental caries infection and biofilm detection for the present study was high in the study area. The predominantly isolated bacterium was *Streptococcus mutans. S. mutans* and *S. aureus* showed high multidrug resistance toward commonly used antibiotics and most of the bacteria isolates were found to be sensitive to cefoxitin and chloramphenicol. Based on our findings, we recommend the integration of routine culture and AST into clinical practice might support the management of multidrug-resistant pathogens among the study population. The educational status, tooth brushing habits, consumption of sugary food, chat chewing, smoking, mouth rinsing habits, kind of material used for brushing, white spots, gum bleeding, sensation to hot and cold, toothache, chronic diseases, and oral hygiene status were significantly associated with the prevalence of dental caries. It is important to provide health education on how to improve oral health in the study area. The education on proper dietary habits might support the prevention and control of dental caries. Further study is needed to find the other determinant factors of dental caries.

## Figures and Tables

**Table 1:** Sociodemographic characteristics of patients attending dental clinics at ACSH and private dental clinic.

Variables	Frequency, *n* (%)
Sex	
Male	227(53.8)
Female	195 (46.2)
Age group (in years)	
≤18 (children)	100 (23.7)
19–64 (adult)	289 (68.5)
≥65 (elder)	33 (7.8)
Residence	
Urban	304 (72.0)
Rural	118 (28.0)
Occupation	
Employed	65 (15.4)
Unemployed	13 (3.1)
Merchant	52 (12.3)
Student	186 (44.1)
Farmers	52(12.3)
Housewife	54 (12.8)
Educational level	
No formal education	42 (9.9)
Primary education	146 (34.6)
Secondary education	113 (26.8)
Tertiary education	121 (28.7)

**Table 2:** Clinical characteristic frequency of dental caries-suspected patients attending ACSH and private clinics.

Variables	Frequency, *n* (%)
White spot	
Yes	207 (49.1)
No	215 (50.9)
Gum bleeding	
Yes	177 (41.9)
No	245 (58.1)
Sensation of tooth ache	
Yes	224 (53.1)
No	199 (47.2)
Dental trauma	
Yes	95 (22.5)
No	327 (77.5)
History of dental procedure	
Yes	145 (34.4)
No	277 (65.6)
Sensation to cold	
Yes	237 (56.2)
No	185 (43.8)
Sensation to hot	
Yes	229 (54.3)
No	193 (45.7)
History of chronic diseases	
Yes	57 (13.5)
No	365 (86.5)

**Table 3:** Behavioral characteristics of dental caries-suspected patients attending ACSH and private clinics.

Variables	Frequency, *n* (%)
A habit of teeth cleaning	
Yes	351(83.2)
No	71 (16.8)
Kind of material used to clean teeth	
Toothpaste	114 (27.0)
Charcoal	42 (10.0)
Wooden tooth sticks	99 (23.5)
Others	96 (27.2)
Time & frequency of teeth clean	
Before & after a meal	100 (23.7)
Twice a day	89 (21.1)
Once a day	88 (20.9)
Once a week	74 (17.5)
Techniques of cleaning teeth	
Top to bottom	84 (19.9)
Sideway	94 (22.3)
Circular	95 (22.5)
Mixed	78 (18.5)
Mouth rising habit	
Yes	353(83.6)
No	69 (16.4)
A habit of sweet food eating/intake	
Yes	351 (83.2)
No	71 (16.8)
Kind of sweet intake	
Sugared coffee	34 (8.1)
Chewing gum containing sugar	42 (10.0)
Candy	61 (14.5)
Chocolate	105 (24.9)
Others (burger, biscuit)	64 (15.2)
Soft drink	
Yes	347 82.2)
No	75 (17.8
Smoking cigarette	
Yes	53 (12.6)
No	369 (87.4)
Chewing chat	
Yes	78 (18.5)
No	344 (81.5)

**Table 4:** Prevalence of bacteria among dental caries-suspected patients attending at ACSH and union private dental clinics regarding sociodemographic 2020.

Variables	Bacterial isolates, *n* (%)			
	*S. mutans* (*n* = 196)	*S. aureus* & *S. mutans* (*n* = 69)	CoNS + S. *mutans* (*n* = 62)	CoNS, *S. aureus*, & *S. mutans* (*n* = 29)
Residence				
Urban	141 (46.4)	62 (41.7)	48 (41.7)	5 (4.3)
Rural	55 (46.6)	7 (53.9)	14 (31.1)	24 (53.3)
Sex				
Male	105 (46.3)	42 (43.8)	33 (34.4)	21 (21.9)
Female	91 (46.7)	27 (42.2)	29 (45.3)	8 (12.5)
Age categories				
≤18	53 (53.0)	13 (24.5)	35 (66.0)	5 (9.4)
19–64	127 (43.9)	55 (57.3)	27 (28.1)	14 (14.6)
≥65	16 (48.5)	1 (9.1)	0 (0)	10 (90.9)
Occupation				
Employed	28 (43.1)	28 (100)	0 (0)	0 (0)
Unemployed	4 (30.8)	4 (100)	0 (0)	0 (0)
Merchant	21 (40.4)	15 (83.3)	0 (0)	3 (16.7)
Student	92 (49.5)	22 (23.9)	62 (67.4)	8 (8.7)
Farmers	27 (51.9)	0 (0)	0 (0)	18 (100)
Housewife	24 (44.4)	0 (0)	0 (0)	0 (0)
Educational level				
No formal education	27 (72.7)	4 (16.7)	1 (8.3)	12 (83.3)
Primary education	74 (50.7)	23 (36.5)	28 (44.4)	12 (19.0)
Secondary education	50 (44.2)	21 (48.8)	20 (46.5)	2 (4.7)
Tertiary education	75 (62.0)	21 (58.3)	13 (36.1)	2 (5.6)

**Table 5:** Univariate and multivariate analysis of the associated risk factors among dental caries-suspected patients attending ACSH and the union private dental clinics.

Variables	Dental caries results		COR (CI 95%)	*P* value	AOR (95%)	*P* value < 0.05
	Positive	Negative				
Age						
≤18	53 (53.0)	47 (47.0)	0.695 (0.441-1.097)	0.118		
19–64	127 (43.9)	162 (56.1)	0.835 (0.380-1.835)	0.653		
≥65	16 (48.5)	17 (51.5)	1			
Sex						
Male	105 (46.3)	122 (53.7)	1		—	—
Female	91 (46.7)	104 (53.3)	1.017 (0.693-1.492)	0.933	—	—
Residences						
Urban	141 (46.4)	163 (53.6)	1			
Rural	55 (46.6)	63 (53.4)	1.009 (0.659-1.546)	0.966	—	—
Educational status						
No formal education	26 (61.9)	16 (38.1)	4.348 (1.097-17.226)	0.59	—	—
Primary education	74 (50.7)	72 (49.3)	1.676 (1.027-2.735)	0.39	—	—
Secondary education	50 (44.2)	63 (55.8)	1.294 (0.768-2.181)	0.333	—	—
Tertiary education	46 (38.0)	75 (62.0)	1			
Occupation						
Employed	28 (43.1)	37 (56.9)	1			
Unemployed	4 (30.8)	9 (69.2)	0.587 (0.164-2.104)	0.414	—	—
Merchant	21 (40.4)	31 (59.6)	0.895 (0.427-1.877)	0.769	—	—
Student	92 (49.5)	94 (50.5)	1.293 (0.732-2.284)	0.376	—	—
Farmers	27 (51.9)	25 (48.1)	1.427 (0.686-2.970)	0.341	—	—
Housewife	24 (44.4)	30 (55.6)	1.057 (0.511-2.188)	0.881	—	—
White spot						
Yes	116 (56.0)	91 (44.0)	2.151 (1.457-3.176)	0.000	3.885 (1.282-11.767)	0.016^∗^
No	80 (37.2)	135 (62.8)	1			
Gum bleeding						
Yes	99 (55.9)	78 (44.1)	1.937 (1.309-2.866)	0.001	2.820 (1.006-7.907)	0.049^∗^
No	97 (39.6)	148 (60.4)	1			
Toothache						
Yes	117 (52.2)	107 (47.8)	1.613 (1.096-2.373)	0.011	2.27 (0.58-0.885)	0.033^∗^
No	79 (39.9)	119 (60.1)	1			
Dental trauma						
Yes	45 (47.4)	50 (52.6)	1.049 (0.664-1.658)	0.42	—	—
No	151 (46.2)	176 (53.8)	1			
Dental procedure						
Yes	70 (48.3)	75 (51.7)	1.119 (0.748-1.673)	0.585	—	—
No	126 (45.5)	151 (54.5)	1			
Sensation to cold						
Yes	114 (48.1)	123 (51.9)	0.859 (0.584-1.264)	0.440		
No	82 (44.3)	103 (55.7)	1			
Sensation to hot						
Yes	109 (47.6)	120 (52.4)	1.873 (1.265-2.773)	0.605		
No	87 (45.1)	106 (54.9)	1			
Do you clean your teeth?						
Yes	163 (46.4)	188 (53.6)	1			
No	3 3(46.5)	38 (53.5)	1.002 (0.601-1.670)	0.995	—	—
If your answer is yes, how often?						
Before & after meal	48 (48.0)	52 (52.0)	1			
Twice a day	36 (40.4)	53 (59.6)	0.736 (0.413-1.311)	0.298	—	—
Once a day	36 (40.9)	52 (59.1)	0.750 (0.421-1.337)	0.330	—	—
Once a week	34 (45.9)	40 (54.1)	0921 (0.504-1.682)	0.788	—	—
Kind of material use						
Tooth paste	45 (39.5)	69 (60.5)	1			
Charcoal	12 (28.6)	30 (71.4)	0.613 (0285-1.321)	0.212	—	—
Wooden tooth sticks	46 (46.5)	53 (53.5)	1.331 (0.772-2.295)	0.304	—	—
Others	51 (53.1)	45 (46.9)	1.738 (1.003-3.010)	0.49	—	—
Type of techniques						
Top to bottom	38 (45.2)	46 (54.8)	1.354 (0.722-2.539)	0.344	—	—
Side way	39 (41.5)	55 (58.5)	1.219 (0.665-2.232)	0.522	—	—
Circular	41 (43.2)	54 (56.8)	1.234 (0.678-2.246)	0.492	—	—
Mixed	36 (46.2)	42 (53.8)	1			
Mouth rinsing habit						
Yes	163 (46.2)	190 (53.8)	1			
No	33 (47.8)	36 (52.2)	1.069 (0.637-1.791)	0.802	—	—
Sweet intake/food						
Yes	180 (51.3)	171 (48.7)	3.618 (1.996-6.559)	0.821		
No	16 (22.5)	55 (77.5)	1			
Kind of sweet intake						
Sugared coffee	12 (35.3)	22 (64.7)	1			
Chewing gum	20 (47.6)	22 (52.4)	1.667 (0.659-4.216)	0.281	1.238 (0.475-3.223)	0.662
Candy	32 (52.5)	29 (47.5)	2.023 (0.852-4.802)	0.110	1.471 (0.598-3.617)	0.400
Chocolate	65 (61.9)	40 (38.1)	2.979 (1.330-6.671)	0.008	5.314 (1.760-16.040)	0.003^∗^
Others (burger, biscuit)	31 (48.4)	33 (51.6)	1.722 (0.731-4.059)	0.214	3.2565 (0.682-15.653)	0.139
Sugared tea	20 (44.4)	25 (55.6)	1.056 (0.640-1.743)	0.413	2.804 (0.568-13.852)	0.206
Soft drink						
Yes	162 (46.7)	185 (53.3)	1986 (1.131-3.486)	0.831		
No	34 (45.3)	41 (54.7)	1			
Smoking cigarette						
Yes	24 (45.3)	29 (34.7)	1.055 (0592-1.881)	0.856	—	—
No	172 (46.6)	197 (53.4)	1			
Chewing chat						
Yes	38 (48.7)	40 (51.3)	1.118(0.684-1.829)	0.656	—	—
No	158 (45.9)	186 (54.1)	1			
Hx of chronic disease						
Yes	29 (50.9)	28 (49.1)	1.228 (0.702-2.147)	0.471	—	—
No	167 (45.8)	198 (54.2)	1			

Hx: history; COR: crude odds ratio; AOR: adjusted odds ratio; CI: confidence interval.

**Table 6:** Antibacterial susceptibility patterns of the isolated bacterial at ACSH and the union private clinics 2020.

Isolated organisms (n)	Patterns	Antimicrobial drugs										
		PE (%)	CIP (%)	CL (%)	Ery (%)	GE (%)	TE (%)	DOX (%)	CAF (%)	SXT (%)	CEFT%	CEF%
*S. mutans* (*n* = 196)	S	32 (16.3)	113 (57.6)	112 (57.1)	103 (52.5)	96 (48.9)	45 (22.9)	103 (52.6)	162 (82.6)	91 (46.4)	121 (61.7)	164 (83.6)
	I	26 (13.3)	53 (27.0)	71 (36.2)	53 (27.0)	77 (39.3)	26 (13.3)	73 (37.2)	28 (14.3)	40 (20.4)	53 (27.0)	26 (13.3)
	R	138 (70.4)	30 (15.3)	13 (6.6)	40 (20.4)	23 (11.8)	125 (63.7)	20 (10.2)	6 (3.1)	65 (33.2)	22 (11.2)	6 (3.1)

*CoNS* (*n* = 62)	S	7 (11.2)	16 (25.8)	35 (56.5)	24 (38.7)	37 (59.7)	15 (24.2)	34 (54.8)	54 (87.1)	16 (25.8)	38 (61.3)	53 (85.5)
	I	4 (6.5)	7 (11.3)	15 (24.1)	20 (32.3)	14 (22.6)	16 (25.8)	20 (32.3)	5 (8.1)	15 (24.1)	18 (29.0)	6 (9.7)
	R	51 (82.2)	39 (62.9)	12 (19.4)	18 (29.0)	11 (17.7)	31 (50.0)	8 (12.9)	3 (4.8)	31 (50)	6 (9.7)	3 (4.8)

*S. aureus* (*n* = 69)	S	6 (8.7)	15 (21.7)	40 (64.5)	19 (27.6)	41 (59.4)	5 (7.2)	32 (46.4)	51 (73.9)	15 (21.8)	34 (49.3)	56 (81.9)
	I	2 (2.9)	5 (7.2)	15 (24.1)	21 (30.4)	21 (30.5)	8 (11.5)	25 (36.2)	12 (17.4)	18 (26.0)	26 (37.7)	4 (5.7)
	R	61 (88.4)	49 (71.0)	14 (20.3)	29 (42.0)	7 (10.1)	56 (81.1)	12 (17.4)	6 (8.7)	36 (52.2)	9 (13.0)	2 (2.9)

Total (327)	S	45 (13.7)	144 (44.0)	187 (57.2)	146 (44.6)	174 (53.2)	65 (19.8)	169 (51.7)	267 (81.6)	122 (37.3)	193 (59.6)	273 (83.5)
	I	32 (9.7)	65 (19.8)	101 (30.8)	94 (28.7)	112 (34.3)	50 (15.3)	118 (36.1)	45 (13.7)	73 (22.3)	97 (29.6)	36 (11.0)
	R	250 (76.5)	118 (36.1)	39 (11.9)	87 (26.6)	41 (12.5)	212 (64.8)	40 (12.2)	15 (4.6)	132 (40.4)	37 (11.3)	11 (3.4)

PE: penicillin; CIP: ciprofloxacin; CL: clindamycin; Ery: erythromycin; GE: gentamicin; TE: tetracycline; CAF: chloramphenicol; DOX: doxycycline; SXT: trimethoprim/sulfamethoxazole; CEFT: ceftriaxone; CEF: cefoxitin; S: sensitive; I: intermediate; R: resistant.

**Table 7:** Multidrug resistance patterns of the isolated Gram-positive bacteria (*n* = 327) at the Ayder Comprehensive Specialized Hospital and union private dental clinic.

Bacterial isolates	Total (%)	Antimicrobial-resistant pattern						
		Ro	R1	R2	R3	R4	≥R5	MDR
*S. mutans*, *n* = 196	196 (59.9)	10 (5.1)	15 (7.6)	30 (15.3)	23 (11.7)	28 (14.3)	10 (10.2)	81 (41.3%)
*S. aureus*, *n* = 69	69 (21.2)	4 (5.8)	9 (13.)	13 (18.8)	6 (8.7)	14 (20.3)	11 (15.9)	27 (39.1)
*CoNS*, *n* = 62	62 (18.9)	12 (19.3)	5 (8.1)	0 (0.0)	4 (6.4)	9 (13.1)	4 (6.5)	24 (38.7)
Total	*327 (100.0)*	*26 (7.9)*	*29 (8.9)*	*33 (10.1)*	*33 (10.1)*	*51 (15.6)*	*25 (7.6)*	*132 (40.4)*

Ro: not resistant; R1: resistant to one; R2: resistant to two; R3: resistant to three, R4: resistant to four; R2: resistant to five antibiotics; MDR: multidrug resistant.

## Data Availability

All the data are available within the manuscript and its supporting information.

## Supplementary Materials

Supplementary Materials Structured questionnaires to collect patient's information. A) Sociodemographic (including age, sex, residence, occupation, and education, B) clinical (including poor oral hygiene, dental trauma, sensitivity to cold, hot, history of chronic disease, and history of dental procedure), and C) behavioral data (including oral hygiene practice, smoking cigarettes, sweet intake, tooth brushing, and chat chewing).
