# Supplementary material for: Bacterial Profile, Antimicrobial Susceptibility Pattern, and Associated Factors among Dental Caries-Suspected Patients Attending the Ayder Comprehensive Specialized Hospital and Private Dental Clinic in Mekelle, Northern Ethiopia
Source: Biomed Res Int. 2022 Oct 17;2022:3463472. doi: 10.1155/2022/3463472 (PMC9592214; doi:10.1155/2022/3463472)
Supplement: Supplementary Materials — Structured questionnaires to collect patient's information. A) Sociodemographic (including age, sex, residence, occupation, and education, B) clinical (including poor oral hygiene, dental trauma, sensitivity to cold, hot, history of chronic disease, and history of dental procedure), and C) behavioral data (including oral hygiene practice, smoking cigarettes, sweet intake, tooth brushing, and chat chewing). [file 3463472.f1.docx]

# Questionnaire English

**Mekelle University College of Health Science Institute of Biomedical Science**

**Department of Microbiology and Immunology**

A questionnaire format was prepared to study the bacterial profile, antibiotic susceptibility pattern, and associated factors among dental caries suspected patients.

These questionnaires have 23 questions and four parts. Read the instruction and fill the following format correctly by making a circle of choice.

**Questionnaires to be filled by study participants.**

Patient MRN______________Study code ____________ phone number____________

Study site___________date _______

**A-Sociodemograpic data**

1. 1. Age __________________?
2. 2. Sex __________________?
3. Residence address A. Urban B.Rural
4. Educational status (your stepfather, guardian or other male adult living with you)

A. No formal education taken B. Primary education

C. Secondary education D. Tertiary education

1. Occupational status? ___________________

**B-Checklist for clinical data for dental caries patients**

1. Presence of poor oral hygiene of the teeth? A.Yes B.No
2. Presence of gum bleeding? A.Yes B.No
3. Have you had a toothache in the last one month? A.Yes B.No
4. Have you had recent dental trauma? A.Yes B.No
5. Have you had recent dental procedures? A.Yes B.No
6. Do you have sensitivity to cold? A.Yes B.No
7. Do you have sensitivity to hot? A.Yes B.No
8. Do you have a history of chronic disease? A.Yes B.No

**C – Oral hygiene and teeth brushing habit**

1. Do you clean your teeth after a meal? A.Yes B.No
2. If your answer is yes? How often do you clean your teeth?

A.Once per a day B.Twice a day

C.Before and after meal A.Once a week_______________

1. What kind of cleaning materials do you use?

A. Wooden toothpicks B.Charcoal

C. Tooth paste D. if other, specify it_______________

1. What types of techniques when you clean your teeth do you use?

A. Top to bottom B.Side way

C. Mixed D.Circular

1. Do you have a mouth-rinsing habit after food? A.Yes B.No

**D - Behavioral factors**

1) Do you have a habit of sweet intake or food frequently? A.Yes B.No

2) If your answer is yes, what kind of sweet intake/food?

A. Coffee with sugar B.Chewing gum C.Candy

D.Chocolate E.others (burger, Biscuit) F.Tea with sugar

3) Do you smoke cigarettes? A.Yes B.No

4) Do you chew chat? A.Yes B.No

5) Do you drink soft drinks frequently? A.Yes B.No

**Thank you very much for your cooperation!**

Witness name _____________________________________sign___________date ________

Data collector’s name________________________________sign___________date________

# 
